# Lomerizine 2HCl inhibits cell proliferation and induces protective autophagy in colorectal cancer via the PI3K/Akt/mTOR signaling pathway

**DOI:** 10.1002/mco2.83

**Published:** 2021-07-15

**Authors:** Xiang‐Peng Tan, Yan He, Yun‐Na Huang, Can‐Can Zheng, Jun‐Qi Li, Qin‐Wen Liu, Ming‐Liang He, Bin Li, Wen‐Wen Xu

**Affiliations:** ^1^ MOE Key Laboratory of Tumor Molecular Biology National Engineering Research Center of Genetic Medicine Institute of Biomedicine College of Life Science and Technology and The First Affiliated Hospital of Jinan University Jinan University Guangzhou China; ^2^ MOE Key Laboratory of Tumor Molecular Biology and Guangdong Provincial Key Laboratory of Bioengineering Medicine National Engineering Research Center of Genetic Medicine Institute of Biomedicine College of Life Science and Technology Jinan University Guangzhou China; ^3^ MOE Key Laboratory of Tumor Molecular Biology and Key Laboratory of Functional Protein Research of Guangdong Higher Education Institutes Institute of Life and Health Engineering College of Life Science and Technology Jinan University Guangzhou China; ^4^ Department of Biomedical Sciences City University of Hong Kong Hong Kong China

**Keywords:** colorectal cancer, drug repurposing, lomerizine 2HCl, PI3K/Akt/mTOR signaling pathway, protective autophagy

## Abstract

Colorectal cancer (CRC) is one of the most common malignancies currently. Despite advances in drug development, the survival and response rates in CRC patients are still poor. In our previous study, a library comprised of 1056 bioactive compounds was used for screening of drugs that could suppress CRC. Lomerizine 2HCl, which is an approved prophylactic drug for migraines, was selected for our studies. The results of in vitro and in vivo assays suggested that lomerizine 2HCl suppresses cell growth and promotes apoptosis in CRC cells. Moreover, lomerizine 2HCl inhibits cell migration and invasion of CRC. RNA sequencing analysis and Western blotting confirmed that lomerizine 2HCl can inhibit cell growth, migration, and invasion through PI3K/AKT/mTOR signaling pathway and induces protective autophagy in CRC. Meanwhile, autophagy inhibition by 3‐methyladenine (3‐MA) increases lomerizine 2HCl‐induced cell apoptosis. Taken together, these results imply that lomerizine 2HCl is a potential anticancer agent, and the combination of lomerizine 2HCl and autophagy inhibitors may serve as a novel strategy to increase the antitumor efficacy of agents in the treatment of CRC.

## Graphical abstract

Lomerizine 2HCl, which is an approved prophylactic drug for migraines, was selected for our studies. In vitro and in vivo functional assays were performed, and the data suggest that lomerizine 2HCl inhibits cell growth and promotes apoptosis in CRC cells. Moreover, lomerizine 2HCl inhibits cell migration and invasion of CRC. RNA sequencing analysis and Western blotting confirmed that lomerizine 2HCl can inhibit cell growth, migration, and invasion via the PI3K/AKT/mTOR signaling pathway and induces protective autophagy in CRC. Meanwhile, autophagy inhibition by 3‐methyladenine (3‐MA) increases lomerizine 2HCl‐induced cell apoptosis. Taken together, these results suggest that lomerizine 2HCl is a potential therapeutic agent, and the combination of lomerizine 2HCl and autophagy inhibitors may serve as a novel strategy to increase the anticancer efficacy of agents in the treatment of CRC.

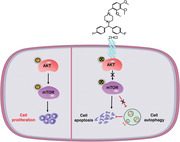

## 1 INTRODUCTION

CRC (colorectal cancer) is a major global disease. In 2018, it was estimated that about 1.8 million new CRC cases were diagnosed, of which nearly 10% resulted in death due to the disease.^1^ Furthermore, 50% of patients initially develop distant metastasis, leading to the high fatality rate.^2^ Current colorectal cancer treatment strategies include surgery, radiotherapy, chemotherapy, cryosurgery, and targeted therapy.^3^ Although a combination of chemotherapy and surgery has generally become the major treatment strategy for colorectal cancer, almost half of CRC patients have a recurrence.^4^ Therefore, exploring novel drugs and the underlying mechanisms of CRC progression are urgently needed.

Drug repurposing is an approach for exploring new applications for drugs that have been approved or are investigational.^5^ Compared to developing a new drug, drug repurposing has many benefits. For instance, the rate of failure is reduced and the time of drug development is shorter.^6^ In the present study, the “drug repurposing” approach was used by employing a small molecule library consisting of 1056 FDA‐approved compounds for initial screening,^7^ and lomerizine 2HCl, which is a prophylactic drug for migraine, was selected for our studies because it showed a high inhibitory effect and it has not been reported to inhibit cancer proliferation according to literature study. We aimed to determine whether lomerizine 2HCl is effective against colorectal cancer, and we illustrate the molecular mechanisms of its effects.

The phosphoinositide 3‐kinase (PI3K)‐AKT pathway is an important activation pathway in cancer.^8^ Activation of the pathway regulates cell metabolism in cancer, thus meeting the growth needs of cancer cells.^9^ The PI3K/AKT pathway is crucial in cell proliferation, apoptosis, and oncogenesis.^10^ Our former studies also reported that the PI3K/AKT signaling is important in esophageal tumor growth and metastasis.^11^ In the present study, lomerizine 2HCl was shown to exert anticancer effects through the PI3K/AKT signaling pathway in CRC.

Autophagy is an intracellular metabolic process in which misfolded proteins, protein aggregates, and damaged organelles are delivered to lysosomes and degraded by autophagosomes.^12^ While excessive autophagy can induce autophagic cell death, autophagy is a protective cellular mechanism during conditions of cellular stress, such as starvation, hypoxia, oxidative stress, and anticancer drug treatment. It has also been shown that autophagy can provide metabolic fuel for tumors and promote tumor progression.^12^ Therefore, exploring the function and mechanisms of protective autophagy in cancer is helpful for developing promising therapeutic strategies to enhance chemotherapy efficacy.

## 2 RESULTS

### 2.1 Lomerizine 2HCl inhibits proliferation and induces apoptosis of CRC cells

**FIGURE 1:**
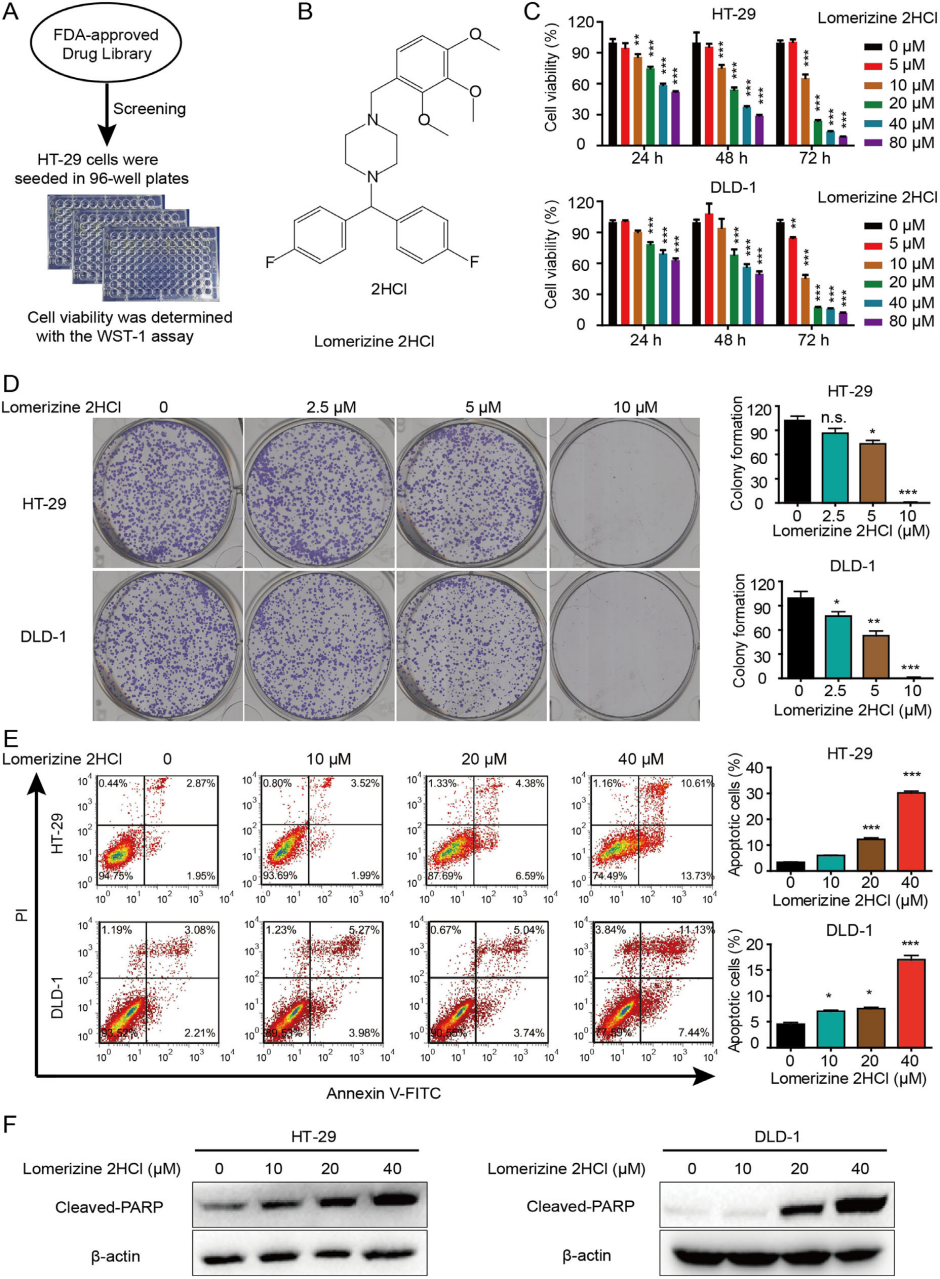
Lomerizine 2HCl inhibits proliferation and induces apoptosis of CRC cells. (A) Diagram shows the screening approach. (B) Chemical structure of lomerizine 2HCl is shown. (C) HT‐29 and DLD‐1 cells were treated with lomerizine 2HCl (5, 10, 20, 40, and 80 μM) for 5 days and the cell viability was detected by CCK‐8 with DMSO as control. (D) Colony formation assay shows the effects of lomerizine 2HCl on colony formation. (E) Flow cytometry analysis shows the effects of lomerizine 2HCl (DMSO, 10, 20, and 40 μM) on cell apoptosis at 48 h. (F) Cells were treated with lomerizine 2HCl for 48 h and the cell lysates were collected to analyze apoptosis‐related proteins. All data shown represent three independent experiments. Bars, SD; *p < 0.05, **p < 0.01, ***p < 0.001

The “drug repurposing” approach was used to screen existing drugs with the potential for inhibiting CRC cell viability by using a small molecule library comprised of 1056 FDA‐approved compounds for the first screening (Figure 1A). Lomerizine 2HCl, a prophylactic drug used for migraines, was selected for our studies (Figure 1B). We intended to detect the inhibitory effects of lomerizine 2HCl on CRC cells. We treated HT‐29 and DLD‐1 cells with different concentrations of lomerizine 2HCl, and cell viability was assessed through the CCK‐8. As shown in Figure 1C, lomerizine 2HCl significantly inhibited CRC cell proliferation. The impact of lomerizine 2HCl on colony formation was also determined. After 14‐day exposure to various concentrations of lomerizine 2HCl, we observed that colony formation was suppressed by lomerizine 2HCl in a dose‐dependent manner in CRC cells (Figure 1D).

Next, we explored the ability of lomerizine 2HCl to induce cell apoptosis in CRC. CRC cells were exposed to lomerizine 2HCl for 48 h. As shown in Figure 1E, lomerizine 2HCl induced apoptosis in both two CRC cell lines. In addition, the results of Western blotting provided evidence that lomerizine 2HCl at a concentration within the range of 10–40 μM can induce the expression of poly ADP‐ribose polymerase (PARP) (Figure 1F). These results imply that apoptosis induced by lomerizine 2HCl is correlated with cysteine proteinase activity.

### 2.2 Lomerizine 2HCl suppresses progression of CRC by inhibiting PI3K/AKT/mTOR signaling pathway

**FIGURE 2:**
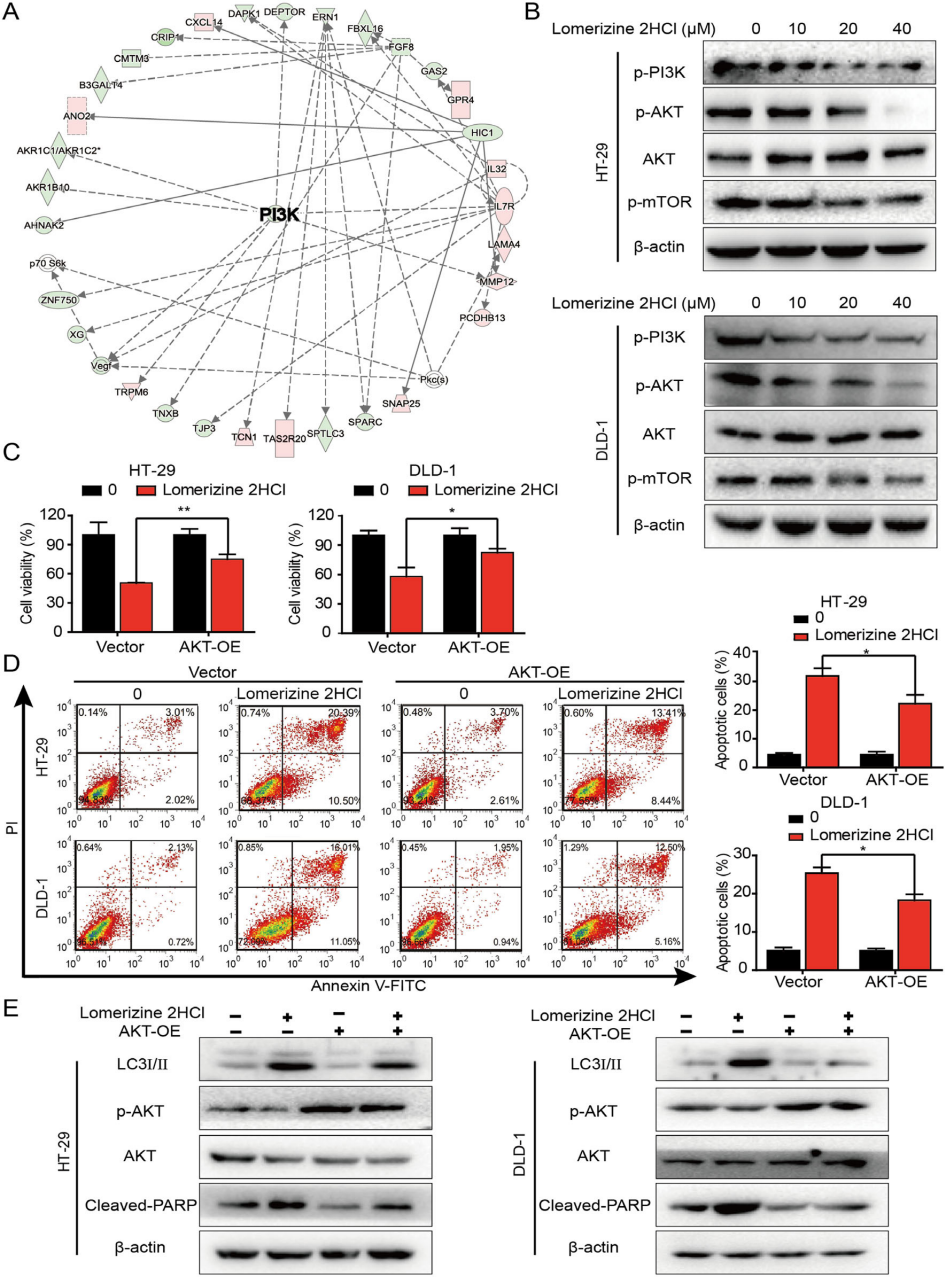
Lomerizine 2HCl suppresses the progression of CRC by inhibiting the PI3K/AKT/mTOR signaling pathway. (A) IPA software has been carried out with RNA‐Seq and identified a significantly altered functional network. (B) CRC cells were incubated with 0, 10, 20, or 40 μM lomerizine 2HCl for 48 h, and the levels of p‐PI3K, AKT, p‐AKT, p‐mTOR, ERK, p‐ERK, MEK, and p‐MEK were detected by Western blot. (C) CRC cells were transfected with AKT plasmid or empty plasmid and then subjected to lomerizine 2HCl (40 μM) treatment. Cell viability was measured by CCK‐8 assay. (D) With the absence or presence of AKT plasmids, CRC cells were treated with lomerizine 2HCl (40 μM), and then cell apoptosis was detected by flow cytometry. (E) Western blot analysis was performed to measure the expression of LC3I/II, AKT phosphorylation, and cleaved PARP in the CRC cells treated with lomerizine 2HCl (20 μM) in the presence or absence of AKT plasmids. Bars, SD; *p < 0.05, **p < 0.01, ***p < 0.001

To explore the molecular mechanisms of lomerizine 2HCl anticancer effects, RNA sequencing was conducted to compare the gene profiles of control and lomerizine 2HCl‐treated CRC cells. The ingenuity pathway analysis (IPA) suggests that lomerizine 2HCl may regulate CRC cells through PI3K signaling pathway (Figure 2A). Table S1 shows the genes with altered expression in the lomerizine 2HCl‐treated cells versus untreated cells. The Western blotting results show that treatment with lomerizine 2HCl led to reduced expressions of p‐PI3K, p‐AKT, and p‐mTOR (Figure 2B).

We then transfected cells with an AKT plasmid to obtain AKT‐overexpressing cells and then subjected these cells to lomerizine 2HCl treatment. As we expected, in the AKT‐overexpressing CRC cells, lomerizine 2HCl‐induced tumor proliferation defect was partially repaired (Figure 2C). In addition, as shown by Annexin V‐FITC/PI staining, AKT overexpression weakened the effects of lomerizine 2HCl‐induced apoptosis and autophagy (Figure 2D,E). It is worth noting that after AKT‐plasmid transfected, the total level of protein did not change, but the p‐AKT increased (Figure 2E). These results suggest that lomerizine 2HCl suppresses the proliferation of CRC through downregulation of the PI3K/AKT/mTOR signaling pathway.

### 2.3 Blocking autophagy promotes lomerizine 2HCl‐mediated apoptosis in CRC cells

**FIGURE 3:**
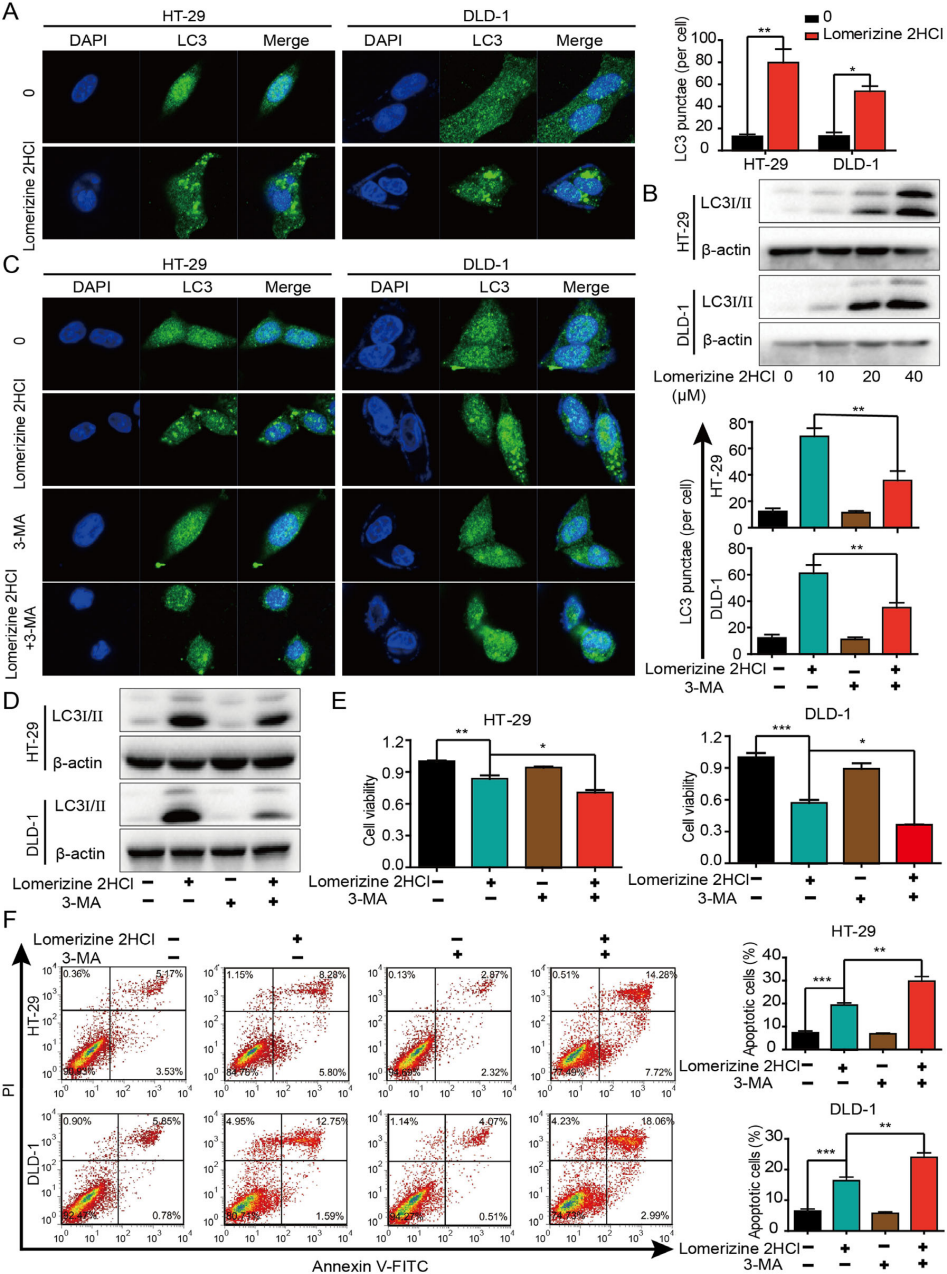
Blocking autophagy promotes lomerizine 2HCl‐mediated apoptosis in CRC cells. (A) CRC cells were treated with or without lomerizine 2HCl (40 μM) for 24 h. Immunofluorescence staining and confocal microscopy imaging show the formation of autophagosomes, indicated by fluorescent‐tagged LC3. (B) CRC cell lines were treated with the indicated concentration of lomerizine 2HCl for 24 h. The expression level of LC3I/II was assessed by Western blot. (C) The formation of autophagosomes detected under the use of autophagy inhibitor (3‐MA, 1 mM) alone and in combination with lomerizine 2HCl (40 μM) is shown. (D) LC3 expression was measured by Western blot. (E) CRC cells were treated with lomerizine 2HCl (40 μM), 3‐MA (1 mM), or the combination of both, and cell viability was assessed by CCK‐8. (F) CRC cells were treated with lomerizine 2HCl (40 μM) or 3‐MA (1 mM) individually or in combination, and cell apoptosis was detected by flow cytometry. Bars, SD; *p < 0.05, **p < 0.01, ***p < 0.001

Autophagy is believed to play an essential role in tumor initiation and growth.^13^ We treated CRC cells with lomerizine 2HCl, and autophagosome accumulation was observed by laser scanning confocal microscopy (Figure 3A). Western blotting analysis also revealed an increase in the expression of LC3I/II with increasing doses of lomerizine 2HCl (Figure 3B). To further verify these results, we used 3‐methyladenine (3‐MA), which is a widely used autophagy inhibitor. As shown in Figure 3C, treatment with lomerizine 2HCl significantly induced autophagosome accumulation than negative control, whereas addition of 3‐MA dramatically reduced the LC3II accumulation induced by lomerizine 2HCl. Western blotting analysis also confirmed these results (Figure 3D). Additionally, CCK‐8 assays revealed that autophagy inhibition by 3‐MA increased the ability of lomerizine 2HCl to suppress tumor proliferation (Figure 3E). Next, we explored the function of autophagy in lomerizine 2HCl‐induced apoptosis. As expected, Annexin V‐FITC/PI assay verified that autophagy inhibition by 3‐MA enhanced CRC cell apoptosis induced by lomerizine 2HCl (Figure 3F). That is, when autophagy was blocked, the oncogenic activities were significantly inhibited. These data show that autophagy suppression may be a promising strategy for anticancer therapy.

### 2.4 Lomerizine 2HCl suppresses migration and invasion in CRC cells

**FIGURE 4:**
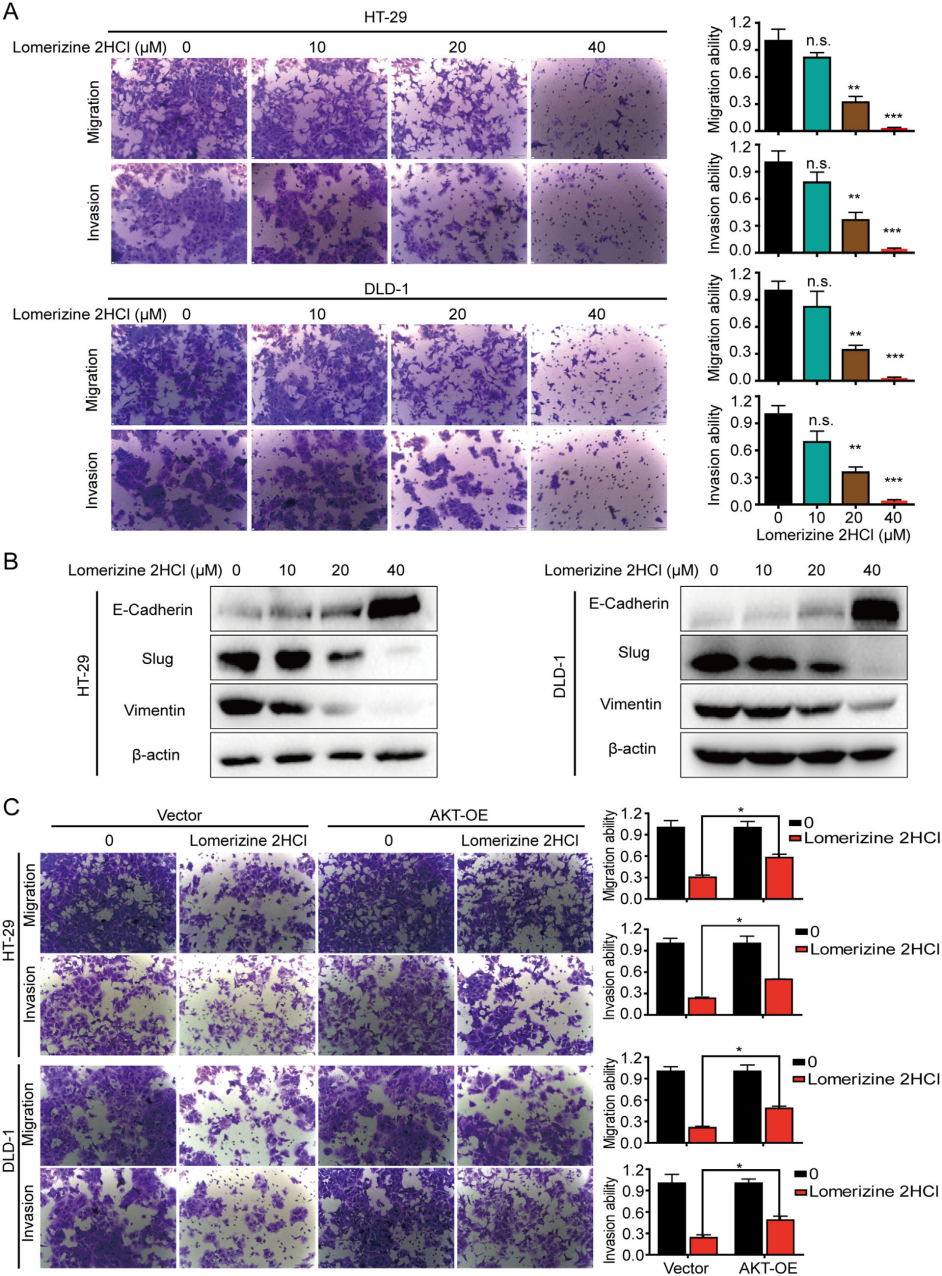
Lomerizine 2HCl suppresses the migration and invasion in CRC cells. (A) Migration and invasion assays show the effects of lomerizine 2HCl on CRC cells (DMSO, 10, 20, 40 μM). (B) CRC cell lines were incubated with increasing doses of lomerizine 2HCl for 48 h, and EMT‐related proteins were detected by Western blot. (C) Migration and invasion ability of HT‐29 and DLD‐1 cells following transfection with AKT‐expressing plasmid or vector control is shown with lomerizine 2HCl treatment (20 μM) for 48 h. The data represent three independent experiments. Bars, SD; *p < 0.05, **p < 0.01, ***p < 0.001

To evaluate inhibitory effect of lomerizine 2HCl on cell migration and invasion, HT‐29 and DLD‐1 cells were exposed to lomerizine 2HCl for 48 h. The migration capacity of both CRC cell types was strongly inhibited by lomerizine 2HCl. In addition, we analyzed whether lomerizine 2HCl could inhibit cell invasion, and the data showed that the invasion of HT‐29 and DLD‐1 cells was significantly suppressed by lomerizine 2HCl (Figure 4A).

Researchers have suggested that the epithelial–mesenchymal transition (EMT) plays a significant role in tumor metastasis.^14^ To illustrate the mechanisms of lomerizine 2HCl‐suppressed CRC cell migration and invasion, we evaluated EMT marker proteins by Western blotting. The results indicate that lomerizine 2HCl dramatically downregulates the expression levels of the vimentin and Slug proteins; however, the levels of E‐cadherin and β‐catenin are elevated (Figure 4B). Collectively, these data imply that lomerizine 2HCl inhibits the migration and invasion of CRC cells by suppressing the EMT process.

To further investigate whether lomerizine 2HCl also inhibits migration and invasion via the PI3K/AKT pathway, CRC cell lines were transfected with an AKT overexpression plasmid. The results show that overexpression of AKT obviously eliminated the inhibitory action of lomerizine 2HCl on cell migration and invasion in CRC cells (Figure 4C).

### 2.5 Synergistic effects of lomerizine 2HCl and 5‐FU on CRC cells

**FIGURE 5:**
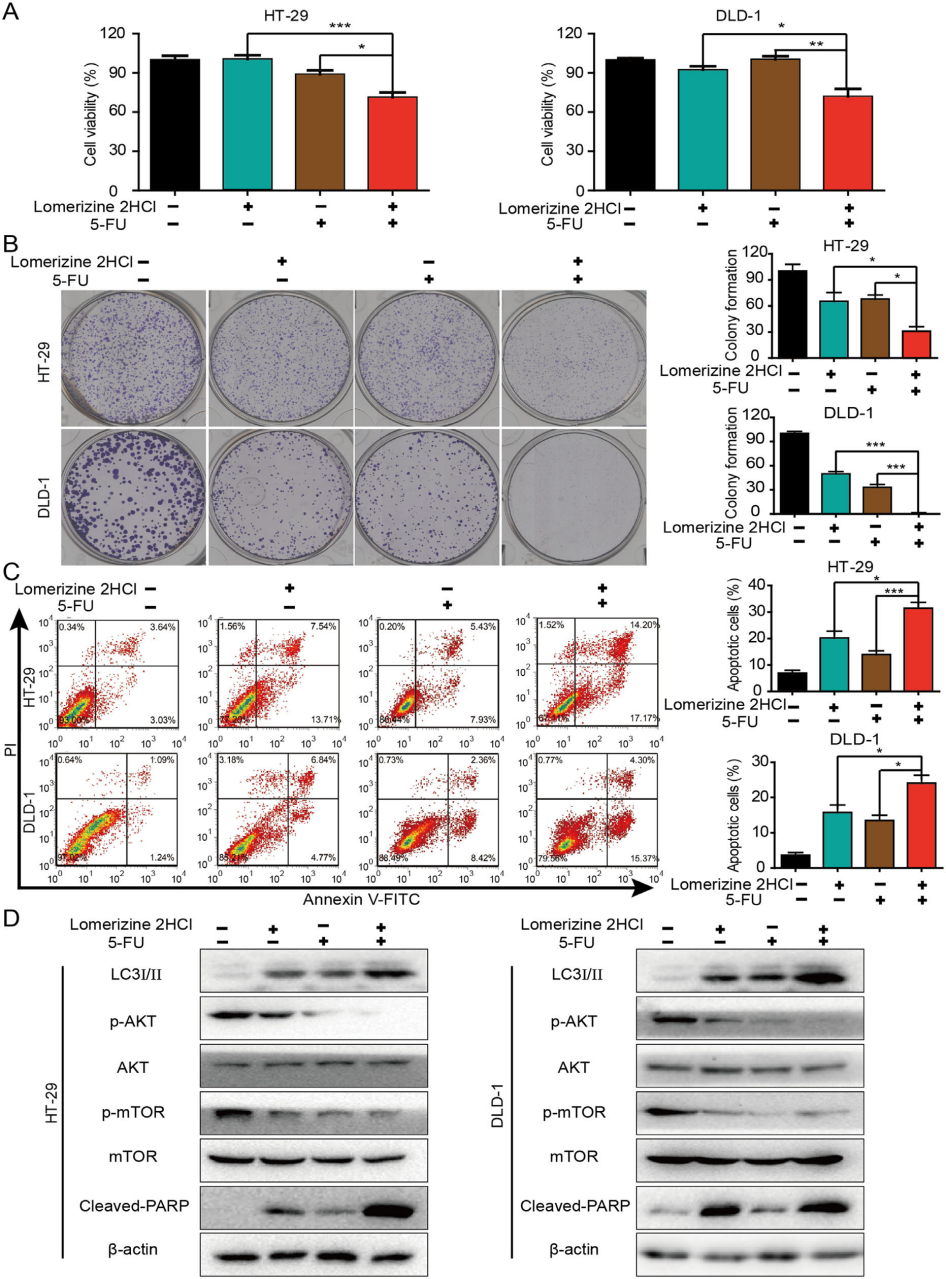
Synergistic effects of lomerizine 2HCl and 5‐FU on CRC cells. (A) Cell viability was assessed by CCK‐8. CRC cells (HT‐29 and DLD‐1) were exposed to lomerizine 2HCl (5 μM), 5‐FU (7.5 μM), or the combination of both. (B) Colony formation assay shows that exposure to lomerizine 2HCl may enhance the drug sensitivity of CRC cells to 5‐FU. (C) Cell apoptosis in CRC cells treated with lomerizine 2HCl (20 μM), 5‐FU (30 μM), or the combination of both is shown. (D) Western blot was carried out to measure the expression of LC3I/II, AKT phosphorylation, mTOR phosphorylation, and cleaved PARP in the CRC cells treated with lomerizine 2HCl (20 μM), 5‐FU (30 μM), or the combination of both. Bars, SD; *p < 0.05, **p < 0.01, ***p < 0.001

Chemotherapy resistance is a major cause of poor prognosis in CRC.^15^, ^16^ The use of drug combinations of several compounds that have different mechanisms of action is another way to make chemotherapy successful.^17^, ^18^ Oral or intravenous 5‐fluorouracil (5‐FU) is the first‐line chemotherapeutic treatment used in CRC.^19^ To determine whether lomerizine 2HCl and 5‐FU have synergistic effects, CRC cells were treated with lomerizine 2HCl and 5‐FU alone or in combination, and the combination of the two drugs significantly inhibited cell viability and colony formation compared with inhibition by either lomerizine 2HCl or 5‐FU alone (Figure 5A,B). To verify the results, we also examined the synergistic effects of lomerizine 2HCl and 5‐FU in tumor cells by flow cytometry and Western blotting analysis. We observed that the combination of lomerizine 2HCl and 5‐FU caused a marked enhancement in the apoptosis of CRC cells than cells treated with lomerizine 2HCl or 5‐FU alone (Figure 5C). Western blotting analysis revealed similar results, as the expression levels of cleaved PARP were elevated in cells exposed to lomerizine 2HCl and 5‐FU (Figure 5D). In addition, our data showed that the combination treatment of lomerizine 2HCl and 5‐FU can effectively suppress AKT pathway. Collectively, the present data show that although lomerizine 2HCl and 5‐FU had slight antiproliferation effects individually, the combination of lomerizine 2HCl and 5‐FU treatment can exert a synergistic effect on inhibiting cell proliferation in tumor cells. Thus, the combination of lomerizine 2HCl and 5‐FU may be an effective therapeutic strategy for colorectal cancer.

### 2.6 Effect of lomerizine 2HCl on tumor growth

**FIGURE 6:**
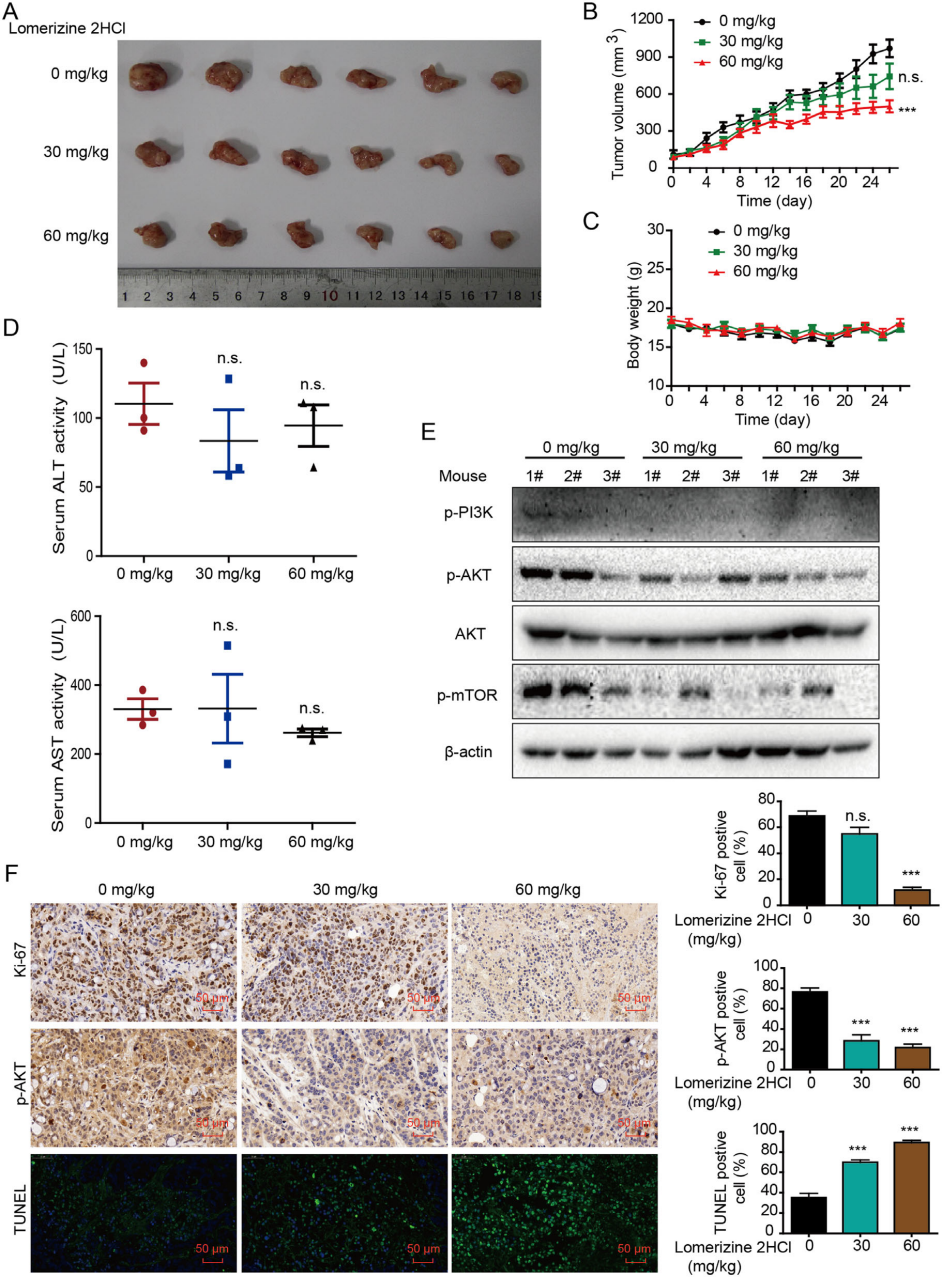
Effect of lomerizine 2HCl on tumor growth. (A) Images show xenografts collected from mice established from HT‐29. (B) Tumor volume curves show that lomerizine 2HCl exerts a significant inhibitory effect on tumor growth (*n* = 6). (C) The bodyweights of mice were monitored. (D) Serum ALT and AST levels in lomerizine 2HCl‐treated mice are compared with vehicle‐treated mice. (E) The expression levels of AKT, p‐AKT, p‐mTOR, and p‐PI3K in lomerizine 2HCl‐treated mice and the vehicle‐treated mice are shown. (F) Images of IHC staining for Ki‐67 and p‐AKT and TUNEL assays are shown in lomerizine 2HCl‐treated mice and vehicle‐treated mice. Bars, SD; *p < 0.05, **p < 0.01, ***p < 0.001

To further confirm the antitumor functions of lomerizine 2HCl in vivo, we established a mouse subcutaneous xenograft model of CRC. When the tumor size reached approximately 100 mm^3^, Balb/c nude mice were separated into three groups randomly that were treated every other day with lomerizine 2HCl (30 and 60 mg/kg) or vehicle (DMSO+PBS). The tumor size in the group treated with 60 mg/kg of lomerizine 2HCl was obviously smaller compared to control group (Figure 6A). Similarly, tumor volume in the group treated with 60 mg/kg of lomerizine 2HCl was also smaller compared to control group (Figure 6B). However, results showed that lomerizine 2HCl treatment did not induce obvious weight loss in the mice (Figure 6C). More importantly, as shown in Figure 6D, changes of alanine aminotransferase (ALT) and aspartate aminotransferase (AST) activity were not obvious in the lomerizine 2HCl group or control group, indicating that lomerizine 2HCl induced only minor damage. In addition, Western blotting analysis of the tumor samples showed that lomerizine 2HCl can significantly downregulate the PI3K/AKT/mTOR signaling pathway in HT‐29 xenografts (Figure 6E). Further, IHC staining revealed lower levels of Ki‐67 and p‐AKT in the lomerizine 2HCl group than in the control, and TUNEL assays indicated higher apoptosis levels in the lomerizine 2HCl group than control group (Figure 6F). In conclusion, above findings suggest that lomerizine 2HCl markedly suppresses the growth of tumor in vivo by suppressing the PI3K/AKT/mTOR signaling pathway, which is consistent with the results observed in vitro.

## 3 DISCUSSION

At present, drug repositioning has gained widespread attention among scientists as a key strategy for accelerating the development of potential anticancer therapies.^20^, ^21^ In this study, based on a chemical library that consisted of FDA‐approved commercial drugs, we found that lomerizine 2HCl exhibited potent anticancer activity against CRC cells. It has been reported that lomerizine 2HCl can decrease the frequency of migraine headaches with an effectiveness of 50%–75%.^22^ However, there have been no studies to investigate the anticancer effects of lomerizine 2HCl. Thus, we provide the first line of evidence showing the suppressive effects of lomerizine 2HCl on cell growth, migration, and invasion. Moreover, we discovered that lomerizine 2HCl can suppress tumor growth in vivo without obvious side effects.

Autophagy can be activated by the stimulation of starvation, hypoxia, oxidative stress, or anticancer drug treatment.^12^, ^23^ According to the literature, autophagy is a double‐edged sword in tumor promotion and metastasis.^24^, ^25^ Autophagy is a conserved process in eukaryotes for the lysosomal degradation of damaged organelles, proteins, and other cytoplasmic components,^26^ but autophagy can also be harmful because it may lead to drug resistance.^27^ In this study, we confirmed that lomerizine 2HCl induced apoptosis in CRC but also triggered protective autophagy. To search for the function of autophagy in experiments, autophagy inhibitor 3‐MA was used in lomerizine 2HCl‐treated CRC cells. We found that lomerizine 2HCl‐induced autophagy was significantly inhibited by 3‐MA, and autophagy suppression increased lomerizine 2HCl‐induced apoptosis in CRC cells. Hence, the combination treatment with lomerizine 2HCl and the autophagy inhibitor enhanced the therapeutic effects of lomerizine 2HCl on CRC cells. Cho has demonstrated that autophagy is associated with the AKT signaling pathway^28^ and that AKT is a positive regulator of cell growth and proliferation.^29^, ^30^ In this study, we found that lomerizine 2HCl suppressed the PI3K/AKT/mTOR signaling pathways in CRC cells. However, AKT overexpression partly reduced the inhibitory effects of lomerizine 2HCl on the AKT/mTOR signaling pathway and weakened the inhibitory actions of lomerizine 2HCl on CRC cell growth. In addition, we found that AKT overexpression could inhibit lomerizine 2HCl‐induced autophagy and weakened lomerizine 2HCl‐induced apoptosis.

CRC is known as a public health problem worldwide because of its high incidence and mortality rate.^31^ Adjuvant chemotherapy is the main treatment strategy for advanced CRC,^32^ and 5‐FU and oxaliplatin are the major chemotherapeutic agents that are used.^33^, ^34^ However, drug resistance develops in patients under long‐term antitumor treatment, and the effectiveness of chemotherapy is often limited.^35^, ^36^ In this study, our results demonstrate that lomerizine 2HCl promotes protective autophagy and suppresses CRC cell growth, migration, and invasion via the PI3K/AKT/mTOR signaling pathway. Based on the advantages reflected in our experimental data, lomerizine 2HCl may become a potential antitumor drug, and suppression of protective autophagy induced by lomerizine 2HCl using 3‐MA might present an effective strategy to improve chemotherapy in CRC.

## 4 MATERIALS AND METHODS

### 4.1 Cell lines and cell culture

The human CRC cell lines (HT‐29 and DLD‐1) were obtained from the Cell Bank of the Chinese Academy of Sciences (Shanghai, China) and cultured in RPMI 1640 medium (Invitrogen, CA, USA) supplemented with 10% fetal bovine serum (FBS; HyClone, UT, USA) at 37°C in 5% CO_2_.

### 4.2 Drugs and plasmids

Lomerizine 2HCl, 5‐FU, and autophagy inhibitor 3‐MA were obtained from Selleck Chemicals (Shanghai, China). The AKT1 (T308D/S473D) plasmid was obtained from Dr. Robert Weinberg. AKT or negative control was transfected into cells with PEI (Polysciences, Inc. USA) reagent (1 mg/ml). Briefly, cells were treated for 4 h with PEI/DNA complex (PEI:DNA = 3:1) diluted by the appropriate serum‐free medium, and then were provided with complete medium.

### 4.3 Cell viability assay

Cell viability was assessed by the Cell Counting Kit‐8 (CCK8) (Mashiki‐machi, Japan) as described previously.^37^ CRC cells were planted in 96‐well plates and exposed to lomerizine 2HCl or 5‐FU at different concentrations for 0–5 days, CCK‐8 was added at the end of treatment. The absorbance at 450 nm was measured on a 96‐well plate reader.

### 4.4 Colony formation assay

The assay was carried out as described previously.^38^ CRC cells were seeded in six‐well plates and treated with assorted concentrations of drugs, followed by culturing for 14 days. After washing, cells were fixed in methanol, then stained by 0.4% crystal violet. After staining, the colonies were rinsed with water and dried for counting. The colonies containing more than 50 cells were counted.

### 4.5 Migration and invasion assay

The assays were executed as previously described.^39^ Transwell chambers with a pore size of 8 μm (Millipore, MA, USA) were used in migration and invasion assays. In invasion assay, Matrigel (Corning, NY, USA) was added to the chambers before the experiment. After rinsing with PBS, cells were resuspended in the medium without serum as follows: the bottom of the chamber was covered with 600 μl of 1640 medium with 20% serum; and the upper chamber was covered with 200 μl cell suspension and 200 μl of medium with different concentrations of drugs. The chambers were fixed by methanol after 48 h, then stained with crystal violet. After staining, the migrated cells were rinsed with water, then dried for counting. The cells that migrated to the bottom chamber were counted using a microscope.

### 4.6 Apoptosis detection by flow cytometry

The experiments were performed as previously described.^40^ Briefly, the Annexin V‐FITC/PI apoptosis detection kit (KeyGen, Nanjing, China) was used. According to the instructions, cells were resuspended in 500 μl of binding buffer and incubated with 5 μl Annexin V‐FITC and 5 μl of PI in the dark, the number of apoptotic cells were recorded by flow cytometry with a FACSCalibur (BD Biosciences).

### 4.7 Immunofluorescence staining

The experiments were executed as previously described.^41^ CRC cells were exposed to varied concentrations of lomerizine 2HCl and fixed with methanol. Then, after treating with 0.5% Triton X‐100, cells were blocked with 5% goat serum and treated with primary antibodies overnight at 4°C. Then cells were covered with the appropriate secondary antibodies for 1 h and 4′,6‐diamidino‐2‐phenylindole (DAPI) for 15 min. Then cells were imaged using laser scanning confocal microscope (LSM700, ZEISS).

### 4.8 Western blot

Western blot was performed as previously described.^42^ After 48h incubation with lomerizine 2HCl or RPMI 1640 medium, CRC cells were collected by pancreatic enzyme digestion and centrifugation. The collected cells were lysed on ice in RIPA buffer containing 1% protease inhibitors. Protein extracts were obtained after high‐speed centrifugation and quantification with the BCA protein quantitation kit (Thermo Fisher, Waltham, USA). Equivalent proteins were resolved by SDS‐PAGE gel and the proteins were subsequently moved to PVDF membrane, then the proteins were blocked and treated with primary antibodies overnight at 4°C. After washing by TBST (TBS containing 1% Tween), appropriate secondary antibodies were added to the membrane to incubate for 60 min, and the bands were observed through an ECL Western blotting kit. The EMT antibody kit, cleaved PARP, p‐AKT (Ser 473), AKT, p‐ERK (Thr202/Tyr204), ERK, p‐PI3K (p85 [Tyr458]/p55 [Tyr199]), and p‐mTOR (Ser 2448) primary antibodies were obtained from Cell Signaling Technology (MA, USA).

### 4.9 RNA sequencing and ingenuity pathway analysis

RNA was extracted by HiPure Total RNA Mini Kit (Megen, Guangzhou, China) and it was sent to the Beijing Genomics Institute Tech (Shenzhen, Guangdong, China) for RNA sequencing (RNA‐seq). Differences of ≥1.5 were defined as differential expressions. The IPA software (Ingenuity Systems, Redwood City, CA, USA) was used for pathway analysis.

### 4.10 Animal experiments

All the tumor xenograft experiments were conducted in accordance with the rules and regulations of the Institute of Laboratory Animal Science at Jinan University. Tumor xenograft experiments were performed as previously described.^43^ Briefly, female Balb/c nude mice at approximately 4–6 weeks old were obtained from GemPharmatech Co., Ltd. (Nanjing, Jiangsu, China). HT‐29 cells in PBS were added the same amount of Matrigel (BD Biosciences), and the cells were subcutaneously injected into the flanks of the mice. We recorded the tumor size and mice weight every other day. The mice were randomly divided into the lomerizine 2HCl‐treated group (30 or 60 mg/kg) and control group (PBS) when tumor size reached 100 mm^3^. After 26 days of lomerizine 2HCl treatment, the mice were euthanized, and the tumor was collected and immediately fixed or preserved at low temperature for further use.

### 4.11 TUNEL assay

The assay was performed as stated by the manufacturer (Servicebio, Wuhan, China) to detect cell death. After deparaffinization and antigen retrieval, the tumor sections were permeabilized using the working solution (Servicebio, Wuhan, China). Then, the sections were added appropriate amount of TDT enzyme and the dUTP mixture at 37°C for 2 h. Finally, sections were stained by DAPI (Servicebio, Wuhan, China) and coverslipped with anti‐fade mounting medium (Servicebio, Wuhan, China). The stained sections were imaged by fluorescence microscope (Nikon Eclipse C1). The exposure settings were adjusted to minimize oversaturation.

### 4.12 Immunohistochemistry

Immunohistochemistry analysis was performed as previously described.^44^ After antigen retrieval and blocking by goat serum, the slides were added with Ki67 antibody (Cell Signaling Technology, 9449) or p‐AKT antibody (Cell Signaling Technology, 4060) overnight at 4°C. After washing, the slides were incubated with horseradish peroxidase (HRP)‐linked secondary antibodies of a universal two‐step test kit (BOSTER, SV002), and the color was visualized with DAB (Servicebio, Wuhan, China). In the end, the slides were stained with hematoxylin and sealed with neutral resin. The stained sections were imaged using fluorescence microscopy (Nikon Eclipse C1). The exposure settings were adjusted to minimize oversaturation.

### 4.13 Statistical analysis

All measurement data were expressed as the mean ± SD and were analyzed by GraphPad Prism software (San Diego, CA, USA) with Student's *t*‐test. We regarded *p*‐values <0.05 as statistically significant.

## CONFLICT OF INTEREST

Ming‐Liang He is the editorial board member of MedComm, but was not involved in the journal's review of, or decisions related to, this manuscript. The other authors declare no conflict of interest.

## ETHICS APPROVAL

The animal experiments were approved by the Animal Experimental Ethics Committee of Jinan University. Tumor volume did not exceed 1500 mm^3^ for mice, the maximal tumour size was not exceeded the ethics committee's standard.

## AUTHOR CONTRIBUTIONS

Wen‐Wen Xu and Bin Li conceived and designed the project. Xiang‐Peng Tan, Yan He, and Yun‐Na Huang performed the experiments. Can‐Can Zheng, Jun‐Qi Li, Qin‐Wen Liu, and Ming‐Liang He participated in the scientific discussion and research design. Wen‐Wen Xu wrote and revised the manuscript.

## Supporting information

SUPPORTING INFORMATION

## Data Availability

All data are available from the corresponding authors upon request.

## References

[1] Chong RC , Ong MW , Tan KY . Managing elderly with colorectal cancer. J Gastrointest Oncol. 2019;10:1266‐1273. 31949947 10.21037/jgo.2019.09.04 PMC6954999

[2] Van Cutsem E , Cervantes A , Nordlinger B , et al. Metastatic colorectal cancer: ESMO Clinical Practice Guidelines for diagnosis, treatment and follow‐up. Ann Oncol. 2014;25:iii1‐iii9. 25190710 10.1093/annonc/mdu260

[3] Breugom AJ , Swets M , Bosset J‐F , et al. Adjuvant chemotherapy after preoperative (chemo)radiotherapy and surgery for patients with rectal cancer: a systematic review and meta‐analysis of individual patient data. Lancet Oncol. 2015;16:200‐207. 25589192 10.1016/S1470-2045(14)71199-4

[4] Anitha A , Maya S , Sivaram AJ , et al. Combinatorial nanomedicines for colon cancer therapy. Wiley Interdiscip Rev Nanomed Nanobiotechnol. 2016;8:151‐159. 26061225 10.1002/wnan.1353

[5] Pushpakom S , Iorio F , Eyers PA , et al. Drug repurposing: progress, challenges and recommendations. Nat Rev Drug Discov. 2019;18:41‐58. 30310233 10.1038/nrd.2018.168

[6] Parvathaneni V , Kulkarni NS , Muth A , et al. Drug repurposing: a promising tool to accelerate the drug discovery process. Drug Discov Today. 2019;24:2076‐2085. 31238113 10.1016/j.drudis.2019.06.014 PMC11920972

[7] Hu HF , Xu WW , Li YJ , et al. Anti‐allergic drug azelastine suppresses colon tumorigenesis by directly targeting ARF1 to inhibit IQGAP1‐ERK‐Drp1‐mediated mitochondrial fission. Theranostics. 2021;11:1828‐1844. 33408784 10.7150/thno.48698 PMC7778598

[8] Lawrence MS , Stojanov P , Mermel CH , et al. Discovery and saturation analysis of cancer genes across 21 tumour types. Nature. 2014;505:495‐501. 24390350 10.1038/nature12912 PMC4048962

[9] Hoxhaj G , Manning BD . The PI3K‐AKT network at the interface of oncogenic signalling and cancer metabolism. Nat Rev Cancer. 2020;20:74‐88. 31686003 10.1038/s41568-019-0216-7 PMC7314312

[10] Shukla S , Maclennan GT , Hartman DJ , et al. Activation of PI3K‐Akt signaling pathway promotes prostate cancer cell invasion. Int J Cancer. 2007;121:1424‐1432. 17551921 10.1002/ijc.22862

[11] Li B , Xu WW , Lam AKY , et al. Significance of PI3K/AKT signaling pathway in metastasis of esophageal squamous cell carcinoma and its potential as a target for anti‐metastasis therapy. Oncotarget. 2017;8:38755‐38766. 28418888 10.18632/oncotarget.16333 PMC5503569

[12] Yang Z , Klionsky DJ . Mammalian autophagy: core molecular machinery and signaling regulation. Curr Opin Cell Biol. 2010;22:124‐131. 20034776 10.1016/j.ceb.2009.11.014 PMC2854249

[13] Yang A , Rajeshkumar NV , Wang X , et al. Autophagy is critical for pancreatic tumor growth and progression in tumors with p53 alterations. Cancer Discov. 2014;4:905‐913. 24875860 10.1158/2159-8290.CD-14-0362 PMC4125497

[14] Thiery JP , Sleeman JP . Complex networks orchestrate epithelial‐mesenchymal transitions. Nat Rev Mol Cell Biol. 2006;7:131‐142. 16493418 10.1038/nrm1835

[15] Serna N , Álamo P , Ramesh P , et al. Nanostructured toxins for the selective destruction of drug‐resistant human CXCR4+ colorectal cancer stem cells. J Control Release. 2020;320:96‐104. 31931052 10.1016/j.jconrel.2020.01.019

[16] Marjaneh RM , Khazaei M , Ferns GA , et al. The role of microRNAs in 5‐FU resistance of colorectal cancer: possible mechanisms. J Cell Physiol. 2019;234:2306‐2316. 30191973 10.1002/jcp.27221

[17] Sun W , Sanderson PE , Zheng W . Drug combination therapy increases successful drug repositioning. Drug Discov Today. 2016;21:1189‐1195. 27240777 10.1016/j.drudis.2016.05.015 PMC4907866

[18] Nikanjam M , Liu S , Kurzrock R . Dosing targeted and cytotoxic two‐drug combinations: lessons learned from analysis of 24,326 patients reported 2010 through 2013. Int J Cancer. 2016;139:2135‐2141. 27389805 10.1002/ijc.30262 PMC5096042

[19] Sakatani A , Sonohara F , Goel A . Melatonin‐mediated downregulation of thymidylate synthase as a novel mechanism for overcoming 5‐fluorouracil associated chemoresistance in colorectal cancer cells. Carcinogenesis. 2019;40:422‐431. 30590435 10.1093/carcin/bgy186 PMC6514450

[20] Sleire L , Forde HE , Netland IA , et al. Drug repurposing in cancer. Pharmacol Res. 2017;124:74‐91. 28712971 10.1016/j.phrs.2017.07.013

[21] Yoshida GJ . Therapeutic strategies of drug repositioning targeting autophagy to induce cancer cell death: from pathophysiology to treatment. J Hematol Oncol. 2017;10:67. 28279189 10.1186/s13045-017-0436-9 PMC5345270

[22] Ikeda K , Hanashiro S , Ishikawa Y , et al. Treatment with telmisartan, a long‐acting angiotensin II receptor blocker, prevents migraine attacks in Japanese non‐responders to lomerizine. Neurol Sci. 2017;38:827‐831. 28224326 10.1007/s10072-017-2854-4

[23] Kaushal GP , Shah SV . Autophagy in acute kidney injury. Kidney Int. 2016;89:779‐791. 26924060 10.1016/j.kint.2015.11.021 PMC4801755

[24] White E . The role for autophagy in cancer. J Clin Invest. 2015;125:42‐46. 25654549 10.1172/JCI73941 PMC4382247

[25] Huang F , Wang BR , Wang YG . Role of autophagy in tumorigenesis, metastasis, targeted therapy and drug resistance of hepatocellular carcinoma. World J Gastroenterol. 2018;24:4643‐4651. 30416312 10.3748/wjg.v24.i41.4643 PMC6224467

[26] Amaravadi RK , Thompson CB . The roles of therapy‐induced autophagy and necrosis in cancer treatment. Clin Cancer Res. 2007;13:7271‐7279. 18094407 10.1158/1078-0432.CCR-07-1595

[27] Wang Y , Bonavida B . A new linkage between the tumor suppressor RKIP and autophagy: targeted therapeutics. Crit Rev Oncog. 2018;23:281‐305. 30311561 10.1615/CritRevOncog.2018027211 PMC6370048

[28] Cho CH . Frontier of epilepsy research ‐ mTOR signaling pathway. Exp Mol Med. 2011;43:231‐274. 21467839 10.3858/emm.2011.43.5.032 PMC3104248

[29] Hammerman PS , Fox CJ , Birnbaum MJ , et al. Pim and Akt oncogenes are independent regulators of hematopoietic cell growth and survival. Blood. 2005;105:4477‐4483. 15705789 10.1182/blood-2004-09-3706 PMC1895036

[30] Song Y , Guo B , Ma S , et al. Naringin suppresses the growth and motility of hypertrophic scar fibroblasts by inhibiting the kinase activity of Akt. Biomed Pharmacother. 2018;105:1291‐1298. 30021366 10.1016/j.biopha.2018.06.103

[31] Feng W , Gong H , Wang Y , et al. CircIFT80 functions as a ceRNA of miR‐1236‐3p to promote colorectal cancer progression. Mol Ther Nucleic Acids. 2019;18:375‐387. 31648103 10.1016/j.omtn.2019.08.024 PMC6819894

[32] Yan J , Dou X , Zhou J , et al. Tubeimoside‐I sensitizes colorectal cancer cells to chemotherapy by inducing ROS‐mediated impaired autophagolysosomes accumulation. J Exp Clin Cancer Res. 2019;38:353. 31412953 10.1186/s13046-019-1355-0 PMC6694658

[33] Yu X , Li Z , Yu J , et al. MicroRNAs predict and modulate responses to chemotherapy in colorectal cancer. Cell Prolif. 2015;48:503‐510. 26202377 10.1111/cpr.12202 PMC6496401

[34] Ab Mutalib NS , Md Yusof NF , Abdul SN , et al. Pharmacogenomics DNA biomarkers in colorectal cancer: current update. Front Pharmacol. 2017;8:736. 29075194 10.3389/fphar.2017.00736 PMC5644034

[35] Kukcinaviciute E , Jonusiene V , Sasnauskiene A , et al. Significance of Notch and Wnt signaling for chemoresistance of colorectal cancer cells HCT116. J Cell Biochem. 2018;119:5913‐5920. 29637602 10.1002/jcb.26783

[36] Hsu HH , Chen MC , Baskaran R , et al. Oxaliplatin resistance in colorectal cancer cells is mediated via activation of ABCG2 to alleviate ER stress induced apoptosis. J Cell Physiol. 2018;233:5458‐5467. 29247488 10.1002/jcp.26406

[37] Xu WW , Zheng CC , Huang YN , et al. Synephrine hydrochloride suppresses esophageal cancer tumor growth and metastatic potential through inhibition of Galectin‐3‐AKT/ERK signaling. J Agric Food Chem. 2018;66:9248‐9258. 30113849 10.1021/acs.jafc.8b04020

[38] Li B , Li J , Xu WW , et al. Suppression of esophageal tumor growth and chemoresistance by directly targeting the PI3K/AKT pathway. Oncotarget. 2014;5:11576‐11587. 25344912 10.18632/oncotarget.2596 PMC4294385

[39] Xu WW , Huang ZH , Liao L , et al. Direct targeting of CREB1 with imperatorin inhibits TGFβ2‐ERK signaling to suppress esophageal cancer metastasis. Adv Sci (Weinh). 2020;7:2000925. 32832354 10.1002/advs.202000925 PMC7435243

[40] Li B , Hong P , Zheng CC , et al. Identification of miR‐29c and its target FBXO31 as a key regulatory mechanism in esophageal cancer chemoresistance: functional validation and clinical significance. Theranostics. 2019;9:1599‐1613. 31037126 10.7150/thno.30372 PMC6485198

[41] Hong P , Liu QW , Xie Y , et al. Echinatin suppresses esophageal cancer tumor growth and invasion through inducing AKT/mTOR‐dependent autophagy and apoptosis. Cell Death Dis. 2020;11:524. 32655130 10.1038/s41419-020-2730-7 PMC7354992

[42] Xu WW , Li B , Guan XY , et al. Cancer cell‐secreted IGF2 instigates fibroblasts and bone marrow‐derived vascular progenitor cells to promote cancer progression. Nat Commun. 2017;8:14399. 28186102 10.1038/ncomms14399 PMC5309924

[43] Xu WW , Li B , Zhao JF , et al. IGF2 induces CD133 expression in esophageal cancer cells to promote cancer stemness. Cancer Lett. 2018;425:88‐100. 29604392 10.1016/j.canlet.2018.03.039

[44] Li B , Xu WW , Guan XY , et al. Competitive binding between Id1 and E2F1 to Cdc20 regulates E2F1 degradation and thymidylate synthase expression to promote esophageal cancer chemoresistance. Clin Cancer Res. 2016;22:1243‐1255. 26475334 10.1158/1078-0432.CCR-15-1196

