# Supplementary material for: Lomerizine 2HCl inhibits cell proliferation and induces protective autophagy in colorectal cancer via the PI3K/Akt/mTOR signaling pathway
Source: MedComm (2020). 2021 Jul 15;2(3):453–66. doi: 10.1002/mco2.83 (PMC8554656; doi:10.1002/mco2.83)
Supplement: Supplementary file 1 — SUPPORTING INFORMATION [file MCO2-2-453-s001.docx]

Table S1 The genes with altered expression in the Lomerizine 2HCl-treated cells vs untreated cells.

| Accession ID | log2FoldChange  (D_1/D_NC) | Up/Down | Accession ID | log2FoldChange  (D_1/D_NC) | Up/Down | Accession ID | log2FoldChange  (D_1/D_NC) | Up/Down |
| --- | --- | --- | --- | --- | --- | --- | --- | --- |
| 29057 | 8.77 | Up | 57655 | -2.08 | Down | 100287216 | -1.27 | Down |
| 101060321 | 7.85 | Up | 22807 | -2.07 | Down | 28514 | -1.26 | Down |
| 103611157 | 7.65 | Up | 23007 | -2.04 | Down | 4600 | -1.26 | Down |
| 728066 | 7.35 | Up | 442038 | -2.04 | Down | 79725 | -1.26 | Down |
| 388436 | 7.01 | Up | 1080 | -2.04 | Down | 83886 | -1.26 | Down |
| 728689 | 6.66 | Up | 440570 | -2.04 | Down | 57449 | -1.26 | Down |
| 54065 | 6.64 | Up | 94122 | -2.04 | Down | 5121 | -1.26 | Down |
| 100996928 | 6.61 | Up | 387036 | -2.04 | Down | 114794 | -1.26 | Down |
| 102724862 | 6.46 | Up | 255394 | -2.04 | Down | 3725 | -1.25 | Down |
| 8293 | 6.43 | Up | 94241 | -2.04 | Down | 28 | -1.25 | Down |
| 267004 | 6.36 | Up | 100381270 | -2.03 | Down | 340485 | -1.25 | Down |
| 96626 | 6.29 | Up | 100271927 | -2.03 | Down | 92104 | -1.25 | Down |
| 100526740 | 5.88 | Up | 84913 | -2.03 | Down | 106865373 | -1.25 | Down |
| 116211 | 5.86 | Up | 2312 | -2.00 | Down | 57568 | -1.25 | Down |
| 375593 | 5.78 | Up | 80129 | -2.00 | Down | 83992 | -1.24 | Down |
| 5342 | 5.58 | Up | 146057 | -2.00 | Down | 100616209 | -1.24 | Down |
| 1270 | 5.55 | Up | 25960 | -2.00 | Down | 9187 | -1.24 | Down |
| 729877 | 5.21 | Up | 65065 | -1.99 | Down | 9056 | -1.24 | Down |
| 54657 | 5.00 | Up | 388633 | -1.98 | Down | 27134 | -1.24 | Down |
| 222967 | 4.95 | Up | 55808 | -1.97 | Down | 55806 | -1.24 | Down |
| 256051 | 4.25 | Up | 25837 | -1.96 | Down | 4137 | -1.24 | Down |
| 101730217 | 4.09 | Up | 728118 | -1.95 | Down | 83594 | -1.23 | Down |
| 2835 | 3.58 | Up | 100288069 | -1.95 | Down | 7450 | -1.23 | Down |
| 101180901 | 3.45 | Up | 2904 | -1.94 | Down | 9037 | -1.23 | Down |
| 401944 | 3.40 | Up | 7429 | -1.93 | Down | 10150 | -1.23 | Down |
| 101928917 | 2.92 | Up | 354 | -1.93 | Down | 64090 | -1.23 | Down |
| 100532731 | 2.63 | Up | 84441 | -1.93 | Down | 288 | -1.23 | Down |
| 348174 | 2.52 | Up | 57644 | -1.92 | Down | 5934 | -1.22 | Down |
| 57101 | 2.29 | Up | 84960 | -1.91 | Down | 6720 | -1.22 | Down |
| 9220 | 2.24 | Up | 11209 | -1.88 | Down | 55040 | -1.22 | Down |
| 100507117 | 2.18 | Up | 6304 | -1.87 | Down | 22941 | -1.21 | Down |
| 3885 | 2.18 | Up | 101927934 | -1.87 | Down | 745 | -1.21 | Down |
| 653268 | 2.14 | Up | 2261 | -1.87 | Down | 286144 | -1.21 | Down |
| 387841 | 2.13 | Up | 26134 | -1.86 | Down | 3081 | -1.21 | Down |
| 378706 | 2.12 | Up | 8705 | -1.85 | Down | 91523 | -1.21 | Down |
| 1815 | 2.06 | Up | 57156 | -1.85 | Down | 7080 | -1.21 | Down |
| 4502 | 2.02 | Up | 81607 | -1.85 | Down | 9666 | -1.21 | Down |
| 83896 | 1.90 | Up | 85442 | -1.84 | Down | 339483 | -1.21 | Down |
| 728 | 1.79 | Up | 1748 | -1.84 | Down | 55304 | -1.20 | Down |
| 9235 | 1.77 | Up | 1800 | -1.83 | Down | 83464 | -1.20 | Down |
| 353514 | 1.72 | Up | 286204 | -1.82 | Down | 221264 | -1.20 | Down |
| 163351 | 1.71 | Up | 129049 | -1.82 | Down | 441194 | -1.20 | Down |
| 6029 | 1.70 | Up | 100288798 | -1.82 | Down | 5362 | -1.20 | Down |
| 100529207 | 1.67 | Up | 25840 | -1.82 | Down | 5797 | -1.20 | Down |
| 2298 | 1.66 | Up | 221472 | -1.81 | Down | 84899 | -1.20 | Down |
| 51200 | 1.65 | Up | 440804 | -1.81 | Down | 100128893 | -1.20 | Down |
| 730755 | 1.65 | Up | 23236 | -1.81 | Down | 54567 | -1.19 | Down |
| 57576 | 1.58 | Up | 388403 | -1.80 | Down | 817 | -1.19 | Down |
| 106182249 | 1.54 | Up | 4776 | -1.79 | Down | 10861 | -1.19 | Down |
| 100527963 | 1.53 | Up | 257019 | -1.79 | Down | 56413 | -1.19 | Down |
| 100507424 | 1.53 | Up | 91464 | -1.78 | Down | 6405 | -1.19 | Down |
| 1305 | 1.53 | Up | 1958 | -1.77 | Down | 5166 | -1.18 | Down |
| 100533483 | 1.53 | Up | 145781 | -1.77 | Down | 220929 | -1.18 | Down |
| 3292 | 1.49 | Up | 30008 | -1.76 | Down | 5365 | -1.18 | Down |
| 283870 | 1.48 | Up | 64798 | -1.76 | Down | 81704 | -1.18 | Down |
| 4493 | 1.47 | Up | 1291 | -1.75 | Down | 80739 | -1.18 | Down |
| 39 | 1.46 | Up | 55616 | -1.75 | Down | 7351 | -1.18 | Down |
| 100996511 | 1.45 | Up | 9249 | -1.75 | Down | 23263 | -1.18 | Down |
| 140894 | 1.45 | Up | 26298 | -1.75 | Down | 8425 | -1.18 | Down |
| 730092 | 1.43 | Up | 102724562 | -1.75 | Down | 155382 | -1.18 | Down |
| 3620 | 1.42 | Up | 64799 | -1.75 | Down | 3892 | -1.18 | Down |
| 399474 | 1.41 | Up | 80054 | -1.74 | Down | 1048 | -1.17 | Down |
| 84541 | 1.38 | Up | 100996712 | -1.73 | Down | 84078 | -1.17 | Down |
| 9214 | 1.36 | Up | 2941 | -1.73 | Down | 57178 | -1.17 | Down |
| 100874123 | 1.35 | Up | 189 | -1.73 | Down | 283314 | -1.17 | Down |
| 84074 | 1.35 | Up | 29943 | -1.72 | Down | 11119 | -1.17 | Down |
| 151295 | 1.35 | Up | 10655 | -1.71 | Down | 87769 | -1.16 | Down |
| 27040 | 1.35 | Up | 392617 | -1.71 | Down | 2921 | -1.16 | Down |
| 101059953 | 1.34 | Up | 3960 | -1.70 | Down | 3554 | -1.16 | Down |
| 4597 | 1.33 | Up | 731275 | -1.70 | Down | 3371 | -1.16 | Down |
| 102723360 | 1.32 | Up | 404201 | -1.70 | Down | 100093631 | -1.16 | Down |
| 401565 | 1.29 | Up | 201229 | -1.69 | Down | 9537 | -1.16 | Down |
| 200315 | 1.26 | Up | 84327 | -1.69 | Down | 54762 | -1.16 | Down |
| 284217 | 1.25 | Up | 6819 | -1.68 | Down | 647166 | -1.16 | Down |
| 346653 | 1.24 | Up | 57016 | -1.68 | Down | 100316868 | -1.15 | Down |
| 6835 | 1.24 | Up | 2081 | -1.68 | Down | 399512 | -1.15 | Down |
| 100113386 | 1.22 | Up | 11093 | -1.67 | Down | 283755 | -1.15 | Down |
| 5271 | 1.21 | Up | 199964 | -1.67 | Down | 7059 | -1.15 | Down |
| 5971 | 1.20 | Up | 90427 | -1.67 | Down | 5333 | -1.15 | Down |
| 441081 | 1.19 | Up | 388588 | -1.66 | Down | 81846 | -1.15 | Down |
| 752014 | 1.15 | Up | 79983 | -1.66 | Down | 84181 | -1.15 | Down |
| 10682 | 1.13 | Up | 79037 | -1.66 | Down | 7498 | -1.15 | Down |
| 64344 | 1.13 | Up | 1843 | -1.65 | Down | 155060 | -1.14 | Down |
| 100128494 | 1.12 | Up | 4151 | -1.65 | Down | 8492 | -1.14 | Down |
| 3931 | 1.10 | Up | 54847 | -1.65 | Down | 10023 | -1.14 | Down |
| 105372480 | 1.09 | Up | 11346 | -1.65 | Down | 100130238 | -1.14 | Down |
| 8637 | 1.08 | Up | 1282 | -1.65 | Down | 715 | -1.14 | Down |
| 653659 | 1.06 | Up | 122622 | -1.64 | Down | 114907 | -1.14 | Down |
| 100861402 | 1.06 | Up | 9122 | -1.63 | Down | 5293 | -1.14 | Down |
| 6352 | 1.06 | Up | 9630 | -1.63 | Down | 5002 | -1.14 | Down |
| 3770 | 1.05 | Up | 150696 | -1.63 | Down | 10825 | -1.14 | Down |
| 3422 | 1.04 | Up | 26471 | -1.62 | Down | 5629 | -1.14 | Down |
| 2810 | 1.04 | Up | 2115 | -1.61 | Down | 8863 | -1.13 | Down |
| 7846 | 1.04 | Up | 643314 | -1.58 | Down | 53840 | -1.13 | Down |
| 100289097 | 1.03 | Up | 100527978 | -1.58 | Down | 10144 | -1.13 | Down |
| 100293516 | 1.03 | Up | 11074 | -1.58 | Down | 80199 | -1.13 | Down |
| 55283 | 1.03 | Up | 64131 | -1.58 | Down | 113263 | -1.13 | Down |
| 442075 | 1.02 | Up | 10913 | -1.58 | Down | 162466 | -1.12 | Down |
| 4501 | 1.01 | Up | 79659 | -1.58 | Down | 79642 | -1.12 | Down |
| 8877 | 1.01 | Up | 283120 | -1.57 | Down | 100131564 | -1.12 | Down |
| 245973 | 1.01 | Up | 130888 | -1.56 | Down | 126375 | -1.12 | Down |
| 84981 | 1.01 | Up | 8537 | -1.55 | Down | 7089 | -1.12 | Down |
| 100302739 | -11.50 | Down | 100132406 | -1.55 | Down | 7133 | -1.12 | Down |
| 728806 | -8.70 | Down | 641298 | -1.55 | Down | 51195 | -1.12 | Down |
| 136319 | -8.62 | Down | 92255 | -1.54 | Down | 100532746 | -1.12 | Down |
| 105369438 | -8.02 | Down | 2212 | -1.54 | Down | 9315 | -1.12 | Down |
| 100534589 | -7.43 | Down | 57597 | -1.54 | Down | 29070 | -1.11 | Down |
| 79999 | -6.93 | Down | 6792 | -1.53 | Down | 93663 | -1.11 | Down |
| 729540 | -6.75 | Down | 101241892 | -1.53 | Down | 80208 | -1.11 | Down |
| 100533952 | -6.64 | Down | 9121 | -1.52 | Down | 1750 | -1.11 | Down |
| 202658 | -6.58 | Down | 388697 | -1.52 | Down | 8864 | -1.11 | Down |
| 100885850 | -6.34 | Down | 161779 | -1.52 | Down | 84532 | -1.10 | Down |
| 100008588 | -6.30 | Down | 8796 | -1.52 | Down | 54414 | -1.10 | Down |
| 386607 | -6.27 | Down | 654463 | -1.51 | Down | 10595 | -1.10 | Down |
| 100529240 | -6.15 | Down | 51655 | -1.50 | Down | 30846 | -1.10 | Down |
| 100861412 | -6.07 | Down | 55777 | -1.50 | Down | 2444 | -1.10 | Down |
| 100532724 | -5.73 | Down | 400618 | -1.50 | Down | 27090 | -1.10 | Down |
| 378108 | -5.55 | Down | 26115 | -1.49 | Down | 2953 | -1.10 | Down |
| 10628 | -5.53 | Down | 255189 | -1.49 | Down | 3169 | -1.09 | Down |
| 57094 | -5.49 | Down | 171389 | -1.49 | Down | 10103 | -1.09 | Down |
| 642402 | -5.13 | Down | 9024 | -1.49 | Down | 197342 | -1.09 | Down |
| 644714 | -5.04 | Down | 100528016 | -1.49 | Down | 57198 | -1.08 | Down |
| 5343 | -5.04 | Down | 9843 | -1.48 | Down | 27113 | -1.08 | Down |
| 392862 | -4.86 | Down | 5337 | -1.48 | Down | 8644 | -1.08 | Down |
| 100529239 | -4.70 | Down | 7043 | -1.48 | Down | 5662 | -1.08 | Down |
| 10451 | -4.52 | Down | 1951 | -1.48 | Down | 7067 | -1.08 | Down |
| 105377924 | -4.52 | Down | 101928483 | -1.48 | Down | 7042 | -1.08 | Down |
| 2984 | -4.46 | Down | 4051 | -1.48 | Down | 22982 | -1.08 | Down |
| 353376 | -4.32 | Down | 79170 | -1.47 | Down | 23321 | -1.08 | Down |
| 647859 | -4.31 | Down | 8416 | -1.47 | Down | 94059 | -1.08 | Down |
| 23017 | -4.25 | Down | 51754 | -1.46 | Down | 3434 | -1.08 | Down |
| 100132287 | -4.25 | Down | 10251 | -1.46 | Down | 9563 | -1.08 | Down |
| 5159 | -4.22 | Down | 23046 | -1.46 | Down | 27148 | -1.08 | Down |
| 2354 | -4.08 | Down | 9884 | -1.46 | Down | 4582 | -1.08 | Down |
| 251 | -3.86 | Down | 9901 | -1.46 | Down | 123099 | -1.07 | Down |
| 8456 | -3.75 | Down | 79800 | -1.45 | Down | 790952 | -1.07 | Down |
| 55321 | -3.70 | Down | 26525 | -1.45 | Down | 158219 | -1.07 | Down |
| 6751 | -3.70 | Down | 100131193 | -1.45 | Down | 221806 | -1.07 | Down |
| 222 | -3.69 | Down | 84614 | -1.44 | Down | 51149 | -1.07 | Down |
| 1469 | -3.64 | Down | 3667 | -1.43 | Down | 124975 | -1.07 | Down |
| 100128385 | -3.58 | Down | 1363 | -1.43 | Down | 113655 | -1.07 | Down |
| 11309 | -3.54 | Down | 23546 | -1.43 | Down | 26153 | -1.07 | Down |
| 1300 | -3.42 | Down | 3680 | -1.43 | Down | 25924 | -1.07 | Down |
| 128864 | -3.41 | Down | 4035 | -1.42 | Down | 201799 | -1.07 | Down |
| 161145 | -3.39 | Down | 100533107 | -1.42 | Down | 7185 | -1.06 | Down |
| 728113 | -3.38 | Down | 3709 | -1.42 | Down | 6337 | -1.06 | Down |
| 22821 | -3.21 | Down | 126823 | -1.42 | Down | 7402 | -1.06 | Down |
| 168544 | -3.14 | Down | 100526836 | -1.42 | Down | 54541 | -1.06 | Down |
| 1159 | -3.13 | Down | 388886 | -1.42 | Down | 286333 | -1.06 | Down |
| 650669 | -3.10 | Down | 55 | -1.41 | Down | 84301 | -1.06 | Down |
| 90668 | -3.09 | Down | 80329 | -1.40 | Down | 57674 | -1.05 | Down |
| 100529144 | -3.05 | Down | 400673 | -1.40 | Down | 10396 | -1.05 | Down |
| 200407 | -3.04 | Down | 360 | -1.40 | Down | 6641 | -1.05 | Down |
| 256158 | -3.00 | Down | 202374 | -1.40 | Down | 84958 | -1.05 | Down |
| 2353 | -2.99 | Down | 200879 | -1.40 | Down | 8831 | -1.05 | Down |
| 6546 | -2.96 | Down | 27092 | -1.39 | Down | 26960 | -1.05 | Down |
| 3936 | -2.95 | Down | 27076 | -1.39 | Down | 100288181 | -1.05 | Down |
| 11240 | -2.93 | Down | 3339 | -1.39 | Down | 8269 | -1.04 | Down |
| 27344 | -2.88 | Down | 125058 | -1.39 | Down | 250 | -1.04 | Down |
| 1646 | -2.88 | Down | 113146 | -1.39 | Down | 100302652 | -1.04 | Down |
| 3783 | -2.87 | Down | 57654 | -1.38 | Down | 6302 | -1.04 | Down |
| 100129931 | -2.81 | Down | 7015 | -1.38 | Down | 124093 | -1.04 | Down |
| 11156 | -2.81 | Down | 55809 | -1.37 | Down | 79660 | -1.04 | Down |
| 54943 | -2.81 | Down | 342979 | -1.37 | Down | 50853 | -1.04 | Down |
| 9619 | -2.78 | Down | 643596 | -1.37 | Down | 9966 | -1.04 | Down |
| 54331 | -2.78 | Down | 727851 | -1.37 | Down | 368 | -1.04 | Down |
| 3394 | -2.78 | Down | 1612 | -1.37 | Down | 4854 | -1.04 | Down |
| 66002 | -2.72 | Down | 100129917 | -1.37 | Down | 80737 | -1.04 | Down |
| 5540 | -2.67 | Down | 6414 | -1.36 | Down | 85014 | -1.04 | Down |
| 9651 | -2.66 | Down | 54097 | -1.36 | Down | 64127 | -1.04 | Down |
| 197257 | -2.66 | Down | 768239 | -1.36 | Down | 5205 | -1.03 | Down |
| 9472 | -2.64 | Down | 57181 | -1.36 | Down | 56667 | -1.03 | Down |
| 91947 | -2.61 | Down | 9022 | -1.35 | Down | 57716 | -1.03 | Down |
| 54084 | -2.49 | Down | 54587 | -1.35 | Down | 5239 | -1.03 | Down |
| 222950 | -2.47 | Down | 8470 | -1.35 | Down | 85446 | -1.03 | Down |
| 5414 | -2.46 | Down | 1803 | -1.35 | Down | 3418 | -1.03 | Down |
| 726 | -2.46 | Down | 4185 | -1.34 | Down | 25849 | -1.02 | Down |
| 100131997 | -2.45 | Down | 101928068 | -1.34 | Down | 727936 | -1.02 | Down |
| 54866 | -2.45 | Down | 275 | -1.34 | Down | 9771 | -1.02 | Down |
| 83715 | -2.44 | Down | 84795 | -1.34 | Down | 5287 | -1.02 | Down |
| 7148 | -2.43 | Down | 57216 | -1.34 | Down | 80724 | -1.02 | Down |
| 4485 | -2.42 | Down | 493 | -1.33 | Down | 7227 | -1.02 | Down |
| 8728 | -2.42 | Down | 79370 | -1.33 | Down | 5268 | -1.02 | Down |
| 644844 | -2.40 | Down | 285386 | -1.33 | Down | 7480 | -1.02 | Down |
| 5087 | -2.39 | Down | 29850 | -1.33 | Down | 333926 | -1.02 | Down |
| 102724594 | -2.36 | Down | 64218 | -1.33 | Down | 5783 | -1.02 | Down |
| 374650 | -2.32 | Down | 9644 | -1.33 | Down | 5118 | -1.01 | Down |
| 23145 | -2.32 | Down | 151887 | -1.32 | Down | 3718 | -1.01 | Down |
| 100505681 | -2.31 | Down | 80022 | -1.32 | Down | 85379 | -1.01 | Down |
| 284697 | -2.29 | Down | 55260 | -1.32 | Down | 80045 | -1.01 | Down |
| 389941 | -2.28 | Down | 151473 | -1.32 | Down | 8844 | -1.01 | Down |
| 23604 | -2.28 | Down | 3203 | -1.32 | Down | 5413 | -1.01 | Down |
| 552891 | -2.27 | Down | 8938 | -1.31 | Down | 63915 | -1.01 | Down |
| 4091 | -2.26 | Down | 55366 | -1.31 | Down | 51196 | -1.01 | Down |
| 401509 | -2.26 | Down | 100506325 | -1.31 | Down | 6712 | -1.01 | Down |
| 170961 | -2.24 | Down | 219699 | -1.31 | Down | 9779 | -1.01 | Down |
| 11005 | -2.23 | Down | 54985 | -1.31 | Down | 4299 | -1.01 | Down |
| 100288695 | -2.20 | Down | 101927881 | -1.31 | Down | 80067 | -1.01 | Down |
| 10411 | -2.19 | Down | 5129 | -1.30 | Down | 84752 | -1.00 | Down |
| 10974 | -2.18 | Down | 23285 | -1.30 | Down | 644943 | -1.00 | Down |
| 148398 | -2.16 | Down | 126567 | -1.30 | Down | 81031 | -1.00 | Down |
| 256714 | -2.16 | Down | 10499 | -1.30 | Down | 65084 | -1.00 | Down |
| 342035 | -2.14 | Down | 146547 | -1.29 | Down | 51090 | -1.00 | Down |
| 80725 | -2.14 | Down | 54760 | -1.28 | Down | 9620 | -1.00 | Down |
| 8794 | -2.13 | Down | 6038 | -1.28 | Down | 84236 | -1.00 | Down |
| 23305 | -2.13 | Down | 92270 | -1.28 | Down | 5577 | -1.00 | Down |
| 6296 | -2.12 | Down | 3077 | -1.28 | Down | 22849 | -1.00 | Down |
| 10223 | -2.12 | Down | 170393 | -1.28 | Down | 55252 | -1.00 | Down |
| 168620 | -2.11 | Down | 8448 | -1.28 | Down | 57458 | -1.00 | Down |
| 399668 | -2.10 | Down | 3209 | -1.27 | Down | 55607 | -1.00 | Down |
| 140688 | -2.10 | Down | 26873 | -1.27 | Down | 414918 | -1.00 | Down |
| 1628 | -2.09 | Down | 101060389 | -1.27 | Down |  |  |  |
| 254827 | -2.09 | Down | 389813 | -1.27 | Down |  |  |  |
